# Recent progress on the detection of animal-derived food stimulants using mass spectrometry-based techniques

**DOI:** 10.3389/fnut.2023.1226530

**Published:** 2023-07-18

**Authors:** Qiang Zhang, Hongying Du, Yingjian Zhang

**Affiliations:** ^1^Graduate School, Capital University of Physical Education and Sports, Beijing, China; ^2^Tangshan Normal University, Tangshan, Hebei Province, China; ^3^Faculty of Sports, Langfang Normal University, Langfang, Hebei Province, China

**Keywords:** chromatography-mass spectrometry, animal-derived food, synthetic steroids, **β**-agonists, zearalenol, glucocorticoids

## Abstract

**Background:**

The misuse of animal-derived stimulants in food is becoming increasingly common, and mass spectrometry (MS) is used extensively for their detection and analysis. There is a growing demand for abused-substances detection, highlighting the need for systematic studies on the advantages of MS-based methods in detecting animal-derived stimulants.

**Objective:**

We reviewed the application of chromatography-mass spectrometry to the screening and detection of food stimulants of animal origin. Specifically, we analyzed four common animal sources of synthetic steroids, β-receptor agonists, zearalenol (ZAL), and glucocorticoids. We also explored the potential of using chromatography-mass spectrometry to detect and analyze animal-derived foods.

**Methods:**

We searched and screened the Web of Science and Google Scholar databases until April 2023. Our inclusion criteria included a publication year within the last 5 years, publication language of English, and the research fields of food analysis, environmental chemistry, and polymer science. Our keywords were “mass spectrometry,” “anabolic androgenic steroids,” “β-2agonists,” “glucocorticoids,” “zearalenone,” and “doping.”

**Results:**

Although traditional techniques such as thin-layer chromatography and enzyme-linked immunoassays are simple, fast, and suitable for the initial screening of bulk products, they are limited by their relatively high detection limits. Among the methods based on MS, gas chromatography–mass spectrometry and liquid chromatography–tandem mass spectrometry are the most widely used for detecting food doping agents of animal origin. However, a sensitive method with high repeatability and a short analysis time for a large number of samples is still required. Advances in MS have enabled the detection of extremely low concentrations of these substances. Combining different techniques, such as high-resolution mass spectrometry, ultra-high performance liquid chromatography–tandem mass spectrometry, gas chromatography-combustion-isotope ratio mass spectrometry, ultra-high performance liquid chromatography-high resolution mass spectrometry, and two-dimensional chromatography, offers significant advantages for detecting trace illicit drugs in animal-derived foods. Due to advances in assay technology and sample preparation methods, sample collection and storage methods such as dried blood spots, dried urine spots, and volumetric absorptive microsampling are increasingly accepted because of their increased stability and cost-effectiveness.

**Significance:**

MS significantly improves the efficiency of detecting doping agents of animal origin. With the continuous development of MS technology, its application in the fields of doping detection and the analysis of doping agents of animal origin is expected to become more extensive.

## 1. Introduction

The use of performance-enhancing drugs (dopants) poses a significant challenge to the healthy development of international sports competitions (1). In an effort to uphold health, ethics, fair play, and honesty in Olympic sports, the World Anti-Doping Agency (WADA) collaborated with several sports organizations to ensure ethical sports actions during the 24th Beijing Winter Olympic Games. The goal was to ensure the fairness of the competition and maintain the physical health of athletes (2). However, some athletes use banned drugs to enhance their performance, which violates the ethics of competitive sports (3), poses considerable harm to their health (4), and in extreme cases, may even lead to death (5). For example, during the 2008 Beijing Olympic Games, the famous Chinese swimmer Ouyang Kunpeng was banned for life for consuming lamb skewers containing clenbuterol (6) Therefore, preventing prohibited drugs from appearing in athletes’ food and ensuring that the food used by athletes complies with regulations are critical for maintaining the fairness of competitions and the health of athletes.

Traditional doping-detection methods suffer from various issues, such as low accuracy, low efficiency, and the requirement for a large number of samples (7). The continuous development of mass spectrometry (MS) has led to its widespread adoption in detecting banned drugs in athletes’ food. MS offers numerous advantages, such as high sensitivity, selectivity, and accuracy, establishing it as the “gold standard” for analyzing abused drugs (8). Utilizing MS can enhance the accuracy and efficiency of detection, promote fairness in sports, safeguard athletes’ health, and facilitate advancements in athlete food testing.

This study reviews the research progress in MS technology applied to food and summarizes the latest developments in the classification, contamination sources, and detection of several common food stimulants. The aim of this review is to raise athletes’ vigilance toward high-risk foods and avoid inadvertently violating anti-doping rules, while providing technical support for food-safety assurance in sports events and theoretical support for the further development of food-borne doping-detection technology.

## 2. Chromatography-mass spectrometry

Chromatography-mass spectrometry is the most widely used and powerful analytical method in food detection and analysis (9). Although chromatography is effective for separating and analyzing mixtures, it is limited in providing information related to the structures of the target substances. In contrast, mass spectrometry ionizes compounds into ions of different masses using electromagnetics and then arranges them into spectra based on their mass-to-charge ratios, thereby providing rich structural information. By combining the fast separation effect of chromatography with the sensitive and accurate analysis capabilities of mass spectrometry, chromatography-mass spectrometry analysis technology enables accurate qualitative and quantitative analysis. Moreover, it can perform unknown screening through full scans and further confirm compounds via secondary mass spectrometry, a spectral library search, ion-fragment analysis, and other methods (9). This technology has become an important screening and detection method in various fields, such as pharmaceutical analysis, food testing, and environmental monitoring, owing to its simplicity, rapidity, high sensitivity, high resolution, and versatility (10–12). Currently, gas chromatography–mass spectrometry (GC–MS), gas chromatography–tandem mass spectrometry (GC–MS/MS), and liquid chromatography–tandem mass spectrometry (LC–MS/MS) are the most commonly used detection methods in chromatography-mass spectrometry analysis, with LC and GC being complementary techniques (13) (Table 1). While the pretreatment steps of LC–MS are relatively simple, its liquid chromatography component has been developed from high-performance liquid chromatography (HPLC) to ultra performance liquid chromatography (UPLC)/ultra high performance liquid chromatography (UHPLC) and from one-dimensional (1D)-LC to 2D-LC (14–16). Chromatography-mass spectrometry is typically divided into low- and high-resolution MS screening methods in screening detection (9) (Supplementary Table S1).

**Table 1:** Comparison of LC–MS/MS and GC–MS for steroid assays (13).

Characteristics	LC–MS/MS	GC–MS
Derivatization	No	Required when the compound is not volatile
Time analysis (speed)	Ten minutes	Thirty minutes
Adaptation to large series	Yes	No
Resolution	Excellent	Good
Applications	Research and clinical research	Research

### 2.1. Low-resolution MS

Triple-quadrupole mass spectrometry (TQMS) is currently the most widely used quantitative-analysis instrument in MS owing to its short analysis time, ability to analyze multiple components, and accurate quantification. However, it is also limited by poor qualitative capabilities in high-resolution and multistage tandem mass spectrometry. TQMS is a low-resolution mass spectrometry technique that includes gas chromatography triple quadrupole mass spectrometry (GC-QQQ-MS), liquid chromatography triple quadrupole mass spectrometry (LC-QQQ-MS), and liquid chromatography quadrupole linear ion trap mass spectrometry (LC-Qtrap-MS). GC-QQQ-MS is primarily suitable for analyzing highly polar and volatile compounds (17). In contrast, triple-quadrupole tandem mass spectrometry can achieve the high-sensitivity identification of complex matrix samples and is suitable for analyzing refractory and thermally unstable compounds, such as pesticides, animal residues, and hormones (18, 19). However, TQMS has certain limitations in terms of selectivity, qualitative ability, and resolution (20). Triple-quadrupole/combined linear ion-trap mass spectrometry combines the selectivity and sensitivity of the series quadrupole with enhanced qualitative function using secondary fragment ions. It utilizes the “trap-trap scanning” mode to generate MRM chromatograms for quantification and MS/MS spectra for qualitative analysis in a single injection. This mode improves secondary fragment ion scanning and significantly enhances the scanning ability of MS. It eliminates false-positive results, enhances the accuracy of qualitative analysis, and improves the qualitative capability for tracing target compounds in complex media.

### 2.2. HRMS

High-resolution mass spectrometry (HRMS) is a powerful technique for collecting full-scan data with high mass accuracy and resolution. It enables the screening and identification of known and unknown compounds with high selectivity and sensitivity (21). Currently, the most commonly used MS type is HRMS, which has a resolution of ≥10, 000 and mass accuracy of less than 5 mg/L (22). The four main types of HRMS are time-of-flight mass spectrometry (TOF-MS), magnetic sector mass spectrometry (MAG-MS), Fourier-transform ion cyclotron resonance mass spectrometry (FT-ICR-MS), and Fourier-transform orbitrap mass spectrometry (FT-Orbitrap MS). These MSs exhibit varying performances owing to differences in their mass-analyzer structures.

#### 2.2.1. TOF-MS

TOF-MS is an MS-based technique developed in the 1990s for substance analysis. The main principle of this technology is that ions with different mass-to-charge ratios, but the same kinetic energy, require different times to pass the same distance in a constant magnetic field (23). This technique provides several advantages, including a wide mass range, fast analysis speed, high sensitivity, high resolution, and accurate mass measurement (achieving a mass accuracy of 5 ppm and resolution of up to 15,000 FWHM) (24). TOF-MS is reliable for qualitatively analyzing trace compounds in complex backgrounds (23). When combined with chromatographic technology, TOF-MS provides an accurate and efficient analytical method for qualitative confirmation, rapidly screening with high throughput, confirming trace compounds with multiple residues, screening unknown compounds, and separating complex matrices. TOF-MS is widely used to analyze known target compounds and screen unknown compounds in food. This technique includes several variations, such as LC-TOF-MS, GC-TOF-MS, LC-Q-TOF-MS, and comprehensive two-dimensional gas chromatography-time-of-flight mass spectrometry (GC × GC-TOF-MS). Combining quadrupole mass spectrometry with TOF-MS can yield accurate masses of quasi-molecular and product ions with higher resolution and better selectivity than TQMS (25).

#### 2.2.2. Electrostatic-field orbitrap mass spectrometry

In 2000, Russian scientist Makarov introduced a novel mass spectrometer known as the “Orbitrap” or electrostatic-field Orbitrap mass spectrometer (26). The Orbitrap provides the advantages of a broad mass range, high mass accuracy, good resolution, high sensitivity, and high analysis speed. It is widely used in trace analyses and provides outstanding advantages in quantitative mass spectrometry (27).

Currently, electrostatic field Orbitrap high-resolution mass spectrometry includes liquid chromatography-linear ion trap electrostatic-field Orbitrap high-resolution mass spectrometry and liquid chromatography-quadrupole electrostatic-field Orbitrap high-resolution mass spectrometry. During the screening process of electrostatic-field Orbitrap high-resolution mass spectrometry, the accurate mass number of the primary mass spectrometry is used for qualitative and quantitative analysis, and fragment ion secondary mass spectrometry is used for further confirmation. Electrostaticfield Orbitrap high-resolution mass spectrometry is used to screen pharmaceuticals, cosmetics, and environmental samples. Liquid chromatography-quadrupole/electrostatic-field Orbitrap high-resolution mass spectrometry has the advantages of high sensitivity, high selectivity, and high mass accuracy, and it is widely used in the fields of pharmaceutical analysis and environmental monitoring (28, 29). The tandem quadrupole electrostatic-field Orbitrap mass spectrometer can scan the compound fully, obtain multilevel mass-spectrometry information of fragment ions, and identify and confirm hundreds of thousands of components in one analysis, which can be accurately quantified (30).

#### 2.2.3. MGA-MS and FT-ICR-MS

MAG-MS is a highly sensitive and stable technique that can achieve resolutions of up to 100,000 pixels (31). Recent advancements in precision machining and fast scanning technologies have improved traditional shortcomings such as poor machining accuracy, instability, and slow scanning speeds. MAG-MS has become more refined and complex and is developing toward higher performance. It is widely used in various industries such as geology, environment, archaeology, semiconductors, food, and life sciences (32). However, the high purchase, operation, and maintenance costs, complicated operation, and slow analysis speed of the instrument make it unsuitable for the routine detection of veterinary-drugs, pesticide residues, and illegal additives.

FT-ICR-MS offers ultra-high resolution and high mass accuracy, but is expensive, bulky, and complicated to operate. Currently, it is only used by a few research laboratories for macromolecular analysis, gas-phase ion reaction kinetics, and complex system analysis.

Low- and high-resolution MS have their own characteristics for sample analysis. High-resolution MS has excellent qualitative advantages and can accurately determine the mass-to-charge ratio of the parent ions. However, when the mass-to-charge ratios of two target substances are similar, accurately characterizing them using LRMS is challenging. Nevertheless, LRMS is highly selective and can easily distinguish between target objects. However, high-resolution MS is easily affected by impurities, and the sample-purification requirements are relatively high. Q-TOF-MS and Orbitrap-MS have been developed for HRMS to detect various drugs. The combination of high-resolution MS and chromatographic technology may lead to the development of high-throughput and high-sensitivity analyses of veterinary-drug residues. TOF-MS can perform qualitative and quantitative analyses and is an effective method for detecting known targets in food samples and screening unknown compounds. The combination of Orbitrap-MS and UPLC is more conducive for the rapid and sensitive screening and detection of unknown compounds in samples. Its ultrahigh resolution ensures the high sensitivity and specificity required for the analysis of complex samples.

## 3. Common doping tests used for food of animal origin

The use of doping in animal husbandry and drug residues in animal-derived foods pose a significant risk to athletes who may consume these products and test positive for doping. The International Olympic Committee (IOC) prohibits doping, and the WADA updates its list of banned substances annually. However, criminals in the animal-husbandry industry continue to use various feed additives and hormonal compounds, such as anabolic steroids, β-receptor agonists, zearalanol, and glucocorticoids, to promote protein synthesis and increase muscle content, even though these substances are banned or restricted in many countries. Positive cases of doping tests mainly arise from drug residues or banned drugs used in animal breeding.

Anabolic steroids, β-2 agonists, glucocorticoids, and zearalenone were the four most commonly detected animal-derived stimulant types (Supplementary Table S2). Detection primarily relied on LC coupled with MS, HPLC coupled with MS, and GC coupled with MS. The use of HRMS, such as Q-TOF, HRMS/MS, and isotope ratio mass spectrometry (IRMS) combined with GC and LC, is becoming the future trend for detection. Most samples were detected in urine, whereas only a few were detected in serum. The types of compounds included in the various stimulants, sample preparation methods, and clean-up methods are listed in the table.

### 3.1. Synthetic steroids

Anabolic androgenic steroids (AASs) are synthetic chemical substances that are structurally and biologically similar to the endogenous male hormone testosterone. AASs are the most common stimulants abused by athletes based on doping testing and are listed under category S1 of the WADA 2020 Prohibited List (Supplementary Table S3). During the 2022 Winter Olympics in Beijing, the Beijing Doping Testing Laboratory reported seven positive cases of doping, including four cases of exogenous AASs involving three substances: dehydrochloromethyltestosterone, mesterolone, and clostebol. AASs enhance protein synthesis, promote muscle growth, improve muscle strength, and increase training intensity and duration, significantly improving athletic performance. However, AASs adversely affect the liver, cardiovascular system, reproductive system, and psychological state. For example, AASs significantly reduce high-density lipoprotein cholesterol, cause testicular atrophy, and impair sperm production and function in male athletes. In female athletes, the effects can be devastating and can include virilization, an enlarged clitoris, increased body hair, and menstrual disturbances. Chronic supraphysiological doses of AASs have also been observed to impair learning and memory ability (33).

**Figure 1:**
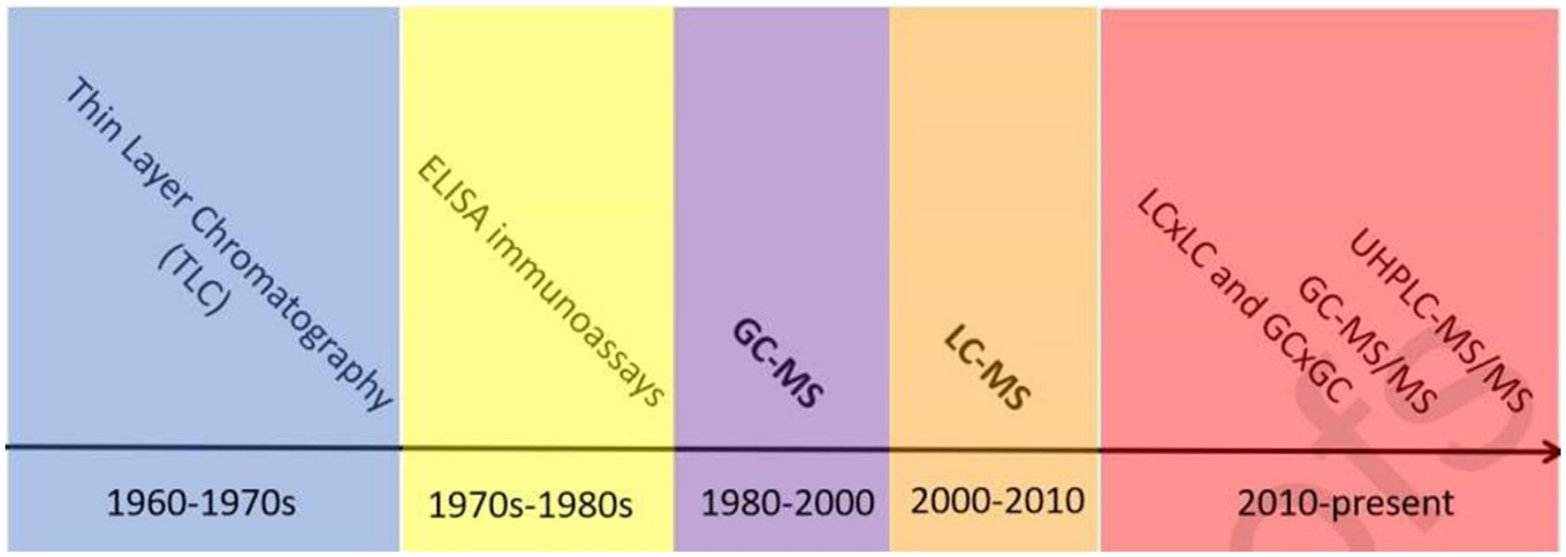
Timeline of steroidal analysis to date, reprinted from (36) with permission.

Over the past few decades, sports drug-monitoring laboratories have made significant progress in detecting AAS residues in various matrices (34, 35) (Figure 1). Methods for detecting synthetic steroids in biological samples include thin-layer chromatography (TLC), enzyme-linked immunosorbent assay (ELISA), HPLC, GC, GC–MS, LC–MS, and LC-tandem mass spectrometry (LC–MS/MS). In the 1960s and early 1970s, TLC with fluorescence detection (TLC-FL) was the most commonly used technique. In the 1970s, immunoassays such as ELISA and enzyme immunoassay were developed and widely used. With improvements in chromatographic separation ability and MS, GC–MS became the mainstream detection method for endogenous anabolic androgenic steroids (EAAS) since the 1980s (37). Owing to the high sensitivity and specificity of GC–MS, the IOC and other international organizations typically use it as the standard method to test for steroid substances. MS was first coupled to GC and then to liquid chromatography. Compared with GC–MS, the pretreatment steps of LC–MS are relatively simple and can compensate for the impact of GC on exogenous proteins with multiple conjugated double-bond structures. The lack of low-sensitivity assays for AASs with high sensitivity and specificity has received extensive attention in recent years. However, the current shift to LC stagnated, and partial shifts to GC were even made, followed by shifts to MS/MS. GC–MS(/MS) is an essential tool for the analysis of saturated steroid metabolites because of their poor ionization (38). While LC and GC techniques have their unique strengths and weaknesses, they can be used in a complementary manner. The decision to use GC or LC depends on the specific compound, matrix, and analytical goals.

UHPLC allows for higher flow rates and a better separation of compounds of similar or equal mass and retention time (such as the α- and β-isomers of testosterone) while reducing run times. Recent studies have demonstrated (39, 40) that UHPLC–MS/MS instruments exhibit excellent detection capabilities for AASs in matrices such as horse plasma and urine. Moreover, LC–MS/MS is now widely used in the detection of anabolic steroids.

HRMS is a mass spectrometry technique with a resolution of 7,500–140,000 pixels or more. It can be used not only for screening, but also for the analyses of biomarkers, such as metabolomics and proteomics (41, 42). Recently, Kaufmann et al. (22) reported the use of HRMS for food analysis, particularly for pesticides and veterinary-drugs. The main advantage of this method is the acquisition of full-scan spectra, which allows compound measurements with compound-specific adjustments, the possibility of retrospective data analysis, and the ability to elucidate the structures of unknown or suspected compounds. With the deepening of ion-source research, many “soft-ionization” techniques combined with GC–MS have been applied in the detection of AASs, including chemical ionization, atmospheric pressure photoionization, and atmospheric pressure chemical ionization. A previous study used gas chromatography-atmospheric-pressure chemical ionization-mass spectrometry to screen 16 exogenous AASs in human urine (43). The results of this study show that atmospheric-pressure chemical ionization produces abundant [M + H]^+^ or [M + H-2TMSOH]^+^ ions, which can be used as precursor ions for ion pairs in the selective ion-monitoring mode, thereby improving the selection of specific substances. Therefore, it can be explained that atmospheric pressure chemical ionization has the potential to replace traditional chemical-ionization sources in the detection of AASs. In addition, the accuracy and sensitivity of the detection instrument significantly improved. Compared with the triple quadrupole, the HRMS has a retrospective full-scan high-resolution data-acquisition mode, which can directly and easily add and verify new unknown compounds. Doping samples stored for 10 years can be used to re-evaluate previously obtained data. Urine was analyzed using gas chromatography-quadrupole-time-of-flight mass spectrometry (GC/Q-TOF) and gas chromatography-tandem quadrupole-electrostatic field orbital ion-trap mass spectrometry (GC/Q-Orbitrap), and the measurement of 46 AASs in human urine was compared using these two instrument types. The analytical performance of the two instruments complied with the WADA specifications, their mass accuracy was excellent, and their detection limits were considerably lower than 50% of the WADA’s minimum required performance level. Both GC–HRMS detection platforms can be used for anti-doping screening. Compared to GC/Q-TOF, the GC/Q-Orbitrap has a higher resolution, sensitivity, and noise-reduction ability (44).

In addition, the development of gas chromatography-carbon combustion-IRMS (GC-C-IRMS) methods capable of distinguishing internal and external sources of some anabolic androgenic steroids and methods for the detection of testosterone esters using a dried blood spot (DBS) have also become a research hotspot in recent years. The use of anabolic steroids, especially the exogenous administration of endogenous steroids such as testosterone, is a significant obstacle for anti-doping regulators. Therefore, doping-control laboratories require analytical methods that can distinguish endogenous steroids from their synthetic analogs in urine. To this end, GC has been used as a highly specialized instrumental-validation technique combined with combustion IRMS to measure the carbon isotope ratio (∆13C) of urinary steroids and determine their synthetic origin based on abnormal ^13^C content (45). Over the past decade, various methods have been developed, and the number of different steroids investigated using IRMS has increased significantly (46). Abuse of norandrosterone, cholerone, corticosteroids, or epitestosterone can also be detected using carbon isotope ratios (47). The use of reference-based values to differentiate endogenous and exogenous isotope ratios is a promising approach. The application of carbon and hydrogen isotope ratios is a fascinating field of study that provides a favorable approach in the fight against doping.

Testing blood samples is one of the most effective strategies for detecting EAAS, and the WADA has approved the analysis of steroid esters in blood matrices. However, whole blood samples have some disadvantages, such as the need for venipuncture and well-trained medical personnel as well as the high cost of transportation and storage (samples need to be stored in a long-term freezer to ensure the stability of serum and plasma) (48). DBS is an alternative matrix with advantages that include minimal invasiveness, cost-effectiveness, and analyte stability (49). Jing et al. (50) have developed a fully automatic online DBS-preparation and detection method based on the DBSA-TLXHRMS system. This method is used for the screening and quantitative analysis of 13 anabolic steroid esters. They successfully verified this method and obtained the extraction recovery of the target compounds from DBS, which ranged from 10.5 to 88.9%. In addition, analyte-stability assessments showed that testosterone undecanoate was more stable (8 weeks) in DBS samples stored under refrigeration (−20°C) than at 4°C. This study demonstrates the suitability of automated DBS analysis for detecting anabolic steroid esters in doping control. The DBS method for detecting anabolic steroids is not currently popular, but it has many advantages and is expected to be widely used in routine doping analyses. Hand et al. (36) considered that WADA-accredited laboratories seemed to pay attention to the improvement of MS, but neglected the improvement of chromatographic technology, especially the application of 2D chromatography. Anti-doping analysis requires extreme accuracy and precision, and although chromatography combined with MS analysis is effective in achieving good selectivity and specificity, there are still some limitations. To meet the minimum performance levels specified by the WADA, laboratories typically use quadrupole mass spectrometry for selective monitoring. This can potentially be cheated by masking agents or shifting peaks because only a small portion of the spectrum is typically used for analysis. Another problem is the inability to detect new or modified compounds or reanalyze samples/spectra. One technique that can be used to overcome these limitations is synthetic two-dimensional chromatography. Compared to conventional separations, GC × GGC enables greater peak capacity (i.e., the number of peaks that can be resolved in a given time) and greater separation of co-eluting compounds, making the technique promising for the complex tasks required for counterdoping. When combined with TOF-MS, this technique has considerable potential and allows full mass-range datasets to be obtained for retrospective analyses. Likewise, LC × LC offers better resolution and can be used either online as part of the analysis or offline as a cleanup step. Two-dimensional analysis requires a similar time to traditional 1D analysis, but combines the 2D-analysis ability to analyze a wider range of samples and separate analytes from the matrix that cannot be detected by 1D analysis (36).

### 3.2. β-agonists

β-receptor agonists are a class of drugs that have a core with a β-phenylethanolamine structure, and they can relax smooth muscles and relieve bronchospasms. β-2 agonists are widely used clinically to treat bronchial asthma; athletes with asthma or exercise-induced asthma must confirm the existence of this condition with the medical committee of their national or international governing body before using β-2 agonist drugs and obtain recognition for therapeutic use exemptions. Non-asthmatic athletes are prohibited from using β-2 agonists as performance-enhancing drugs, which are listed as S3 drugs by WADA 2020. Some athletes and bodybuilders abuse β-agonists to enhance athletic performance and build their body shape. This type of drug can bind to the β-receptor in the cell membrane, thereby producing a series of physiological effects, reducing fat deposition, and increasing the lean-meat percentage (51). Therefore, these drugs are also abused by unscrupulous businesses in food production to increase the yield and lean-meat production rate of meat products, thereby increasing profits. The abuse of drugs may lead to a residue of harmful substances in meat, such as metabolites of β-receptor agonists, which can cause potential harm to human health. Adverse reactions include palpitations, tremors, headaches, dry mouth, and vomiting. Long-term use may also lead to β-receptor downregulation, reduced drug efficacy, and drug resistance.

The detection methods of β-receptor agonists vary according to the sample-matrix pretreatment methods and detection instruments, and they currently include an enzyme-linked immunoassay, radioimmunoassay (RIA), LC, GC–MS/MS, liquid analytical methods such as phase chromatography tandem mass spectrometry, initially ELISA and RIA were used as control methods for β-agonists. These methods use specific antibodies to detect the presence of β-receptor agonists. The advantages of these methods include their rapidity, accuracy, and high sensitivity. However, immunological cross-reactivity can occur, and the quality of the reagents produced by the manufacturer can affect the consistency of the results. Liquid chromatography is only suitable for single-component detection and has disadvantages such as low sensitivity, susceptibility to interference from impurities, and the inability to detect multiple stimulants simultaneously. Consequently, its applications have been limited in recent years.

In comparison, detection institutions tend to use chromatography-mass spectrometry technology with high sensitivity, good repeatability, and strong specificity. The qualitative characteristics of a compound structure can yield better results. Chromatography-mass spectrometry is also a mainstream method for analyzing β-receptor agonists, including gas chromatography–mass spectrometry and liquid chromatography-mass spectrometry. GC–MS analysis was primarily used before the 1980s. Because β-receptor agonists are polar compounds with a high boiling point and low volatility, they are unsuitable for direct GC or GC/MS analysis. First, the sample must be derivatized to increase its volatility and stability. The polarity of the sample is then reduced to improve the analytical effect and increase the sensitivity. This method takes a long time to derivate, the conditions are not well controlled, and there are few receptor agonists that can be detected; thus, the use of this method is decreasing annually. In 1989, Blanchflower and Kennedy used LC–MS for the biological analysis of β-receptor agonists for the first time. As mentioned earlier, the pretreatment of LC–MS/MS is straightforward and does not require derivatization, therefore, results could be obtained quickly. This method is widely used to detect animal-derived β-receptor agonists.

**Figure 2:**
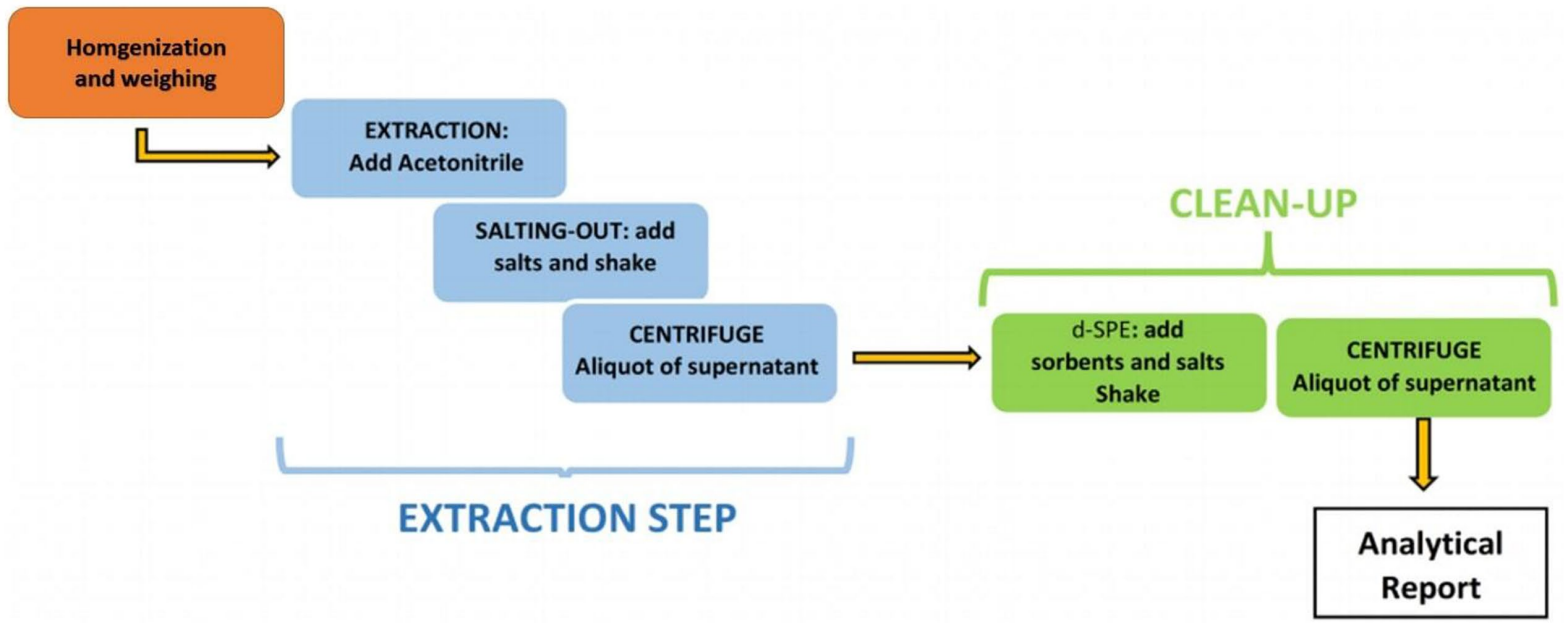
Scheme of standard QuEChERS method, reprinted from (55) with permission.

In recent years, research on the LC–MS detection of β-receptor agonists has focused on the development and application of sample pretreatment and instrumental multi-residue detection technologies. Pretreatment research has mainly focused on the selection of sample hydrolysis and optimization of purification methods. Liu et al. (52) used a 1% trichloroacetic acid solution to ultrasonically extract 26 types of β-receptor agonists from animal tissues for 15 min. Compared to the enzymatic hydrolysis method, the protein function of the analyte and precipitated protein reduces the interference of the enzyme preparation itself and its hydrolyzed products on the target object during enzymatic hydrolysis and meets the requirements of rapid detection. Research on purification-method optimization has mainly focused on solid-phase extraction (SPE). Zhang et al. (53) optimized the sample pretreatment method and applied SPE to HPLC–MS/MS to detect ractopamine, ractopamine, and the albuterol beta-agonist. The detection limits of albuterol and ractopamine were 0.13 and 0.14 μg/kg, respectively, and the relative standard deviations at different substrate concentrations were 5.9–16.2% and 6.4–20.2%, respectively. In the selection of purification methods, purification modes such as online solid-phase extraction and molecular imprinted solid-phase extraction have also made considerable progress by improving working efficiency. However, traditional pretreatment processes, such as SPE and solvent extraction, are relatively complicated, and their detection cycles are long. The quick, easy, cheap, effective, rugged, and safe (QuEChERS) method, a relatively environmentally friendly treatment technology, has been gradually promoted and applied in recent years. Owing to its ability to extract a broad spectrum of analytes, from nonpolar to polar pesticides, the application of this method extends to different analytes outside its classical domain, such as environmental pollutants, amines, and polyphenolic drugs, from different matrices, including food, biological fluids, and environmental samples (54). The QuEChERS method comprises two steps (55) (Figure 2): in the first step, acetonitrile (ACN) is added to the solid matrix, after the addition of salt (usually NaCl or MgCl), between the water and organic layer partitioning is carried out; in the second step, a combination of salt and porous adsorbent is added to the previously obtained ACN solution to remove matrix-interfering substances by dispersive solid-phase extraction (d-SPE). To strictly control harmful substances in food that are risky to human health, the QuEChERS method was applied to better evaluate the contaminants present in meat. Currently, it is used to detect harmful substances such as hormones and β-agonists in meat (56). Oliveira et al. (57) developed a QuEChERS method followed by LC–MS analysis for detecting various pesticides in beef, which was effective for the quantitative determination of more than 150 compounds. The extraction phase consisted of acetonitrile and 1% ethyl acetate (70:30, v:v %), and the washing phase consisted of MgSO4, C18, and PSA.

With the development of detection instruments, the application of ion trap-time-of-flight tandem mass spectrometry has shown obvious advantages, such as high sensitivity, high mass precision analysis of samples, and detection of target compounds in complex samples. Liu et al. (58) established an HPLC-Q-TOF-MS detection method for 31 β-receptor agonists in pork, and the detection limit of 31 compounds was 0.01–5 μg/kg. This was combined with a self-built accurate relative-molecular-mass database, through the comparison of accurate relative molecular mass, retention time, isotope peaks, and characteristic fragment ions, to screen and detect animal-derived foods that may require β-agonists. Other methods for agonist detection include capillary electrophoresis (59) and biosensor methods; however, these methods are imperfect and are rarely used in practical applications because they require further research and development.

### 3.3. Zearalanol

Zearalenone (ZEN) is a type of weak estrogenic mycotoxin that is mainly produced by Fusarium fungi. It belongs to the group of resorcylic acid lactone nonsteroidal assimilation hormones. Taking ZEN can promote muscle contraction, fat breakdown, and metabolism, improve cardiopulmonary function, and enhance the excitability of the nervous system, thereby improving the reaction, endurance, and athletic performance of athletes. This hormone is classified as S1.2, other hormone-like substances, under the WADA’s prohibition list. It is also widely used in animal husbandry to promote animal growth. ZEN can enter the human body through biological accumulation, and it can affect sexual function and the normal development of secondary sexual characteristics. It has certain toxic effects on organs, such as the liver and kidneys, and even exhibits some carcinogenicity. Several countries explicitly prohibit the use of these substances in poultry and livestock farming.

Detection methods for ZEN include TLC, GC, ELISA, fluorescence immunoassay, GC–MS, LC–MS, and LC–MS/MS. TLC and immunoassays are simple, fast, and cost-effective methods; however, they are prone to false positives, making them suitable for the preliminary screening of bulk products. GC requires derivatization, particularly phenolic hydroxyl derivatization, which is complex and rarely used alone. GC–MS can also simultaneously detect multiple substances, but pretreatment requires derivatization, the operation is complex, and personnel quality requirements are high. Owing to the high fluorescence of ZEN, HPLC is widely used for detection owing to its sensitivity, selectivity, and high degree of separation. LC–MS can simultaneously detect multiple components and is reliable for qualitative and quantitative analyses; however, it is susceptible to interference from sample matrices, making quantitative analysis difficult. LC–MS/MS is better suited for the detection of complex matrices, owing to the characteristics of tandem mass spectrometry. It also has the advantages of high throughput and rapid detection, making it the most commonly used method for ZEN detection. Qi et al. (60) used the LC–MS/MS method of the internal standard method to determine the residues of six types of ZEN compounds in animal-derived foods; the simplicity, sensitivity, and stability of the method were all improved. Peter et al. (61) successfully measured the residual ZEN in pig urine, muscle tissue, and liver using LC–MS/MS after the sample was purified via SPE.

HPLC-tandem mass spectrometry (HPLC–MS/MS) is the preferred method for analyzing ZENs owing to its strong separation ability and high sensitivity. However, cumbersome sample-preparation procedures and time-consuming column separations render it unsuitable for rapid detection. Wang et al. (62) proposed a new atmospheric pressure matrix-assisted laser desorption/ionization ion source using a hydrophilic covalent organic framework-coated steel plate (HCOFCS) as the electrospray ionization tip. Based on the synthesis of the HCOFCS, a fast and sensitive AMS method was established to detect trace amounts of ZEN and its derivatives in milk. Compared to traditional LC–MS methods, this method is simple to operate and has a high enrichment factor, high sensitivity, and low cost. This method was used to determine five ZENs and their derivatives in milk samples, including ZEN, α-Zearalenol, β-Zearalenol, α-Zearalanol, and β-Zearalanol. The results showed that HCOFCS combined with ESI-MS could rapidly and sensitively determine trace amounts of ZEN and its derivatives in milk samples, with recoveries ranging from 80.58 to 109.98% and good repeatability. The study also showed that the HCOFCS has good reusability and can be reused at least ten times with good adsorption performance.

GC and HPLC typically require high-purity solvents, which may limit their application. Therefore, high-performance thin-layer chromatography (HPTLC) is an effective method. It can simultaneously separate and identify multiple samples in a short time and is an inexpensive, simple, reliable, and efficient method. HPTLC can be combined with MS, which enables the rapid confirmation of analytes in samples. HPTLC methods for determining ZEN in grains are available worldwide (63). Jorquera et al. (64) established an HPTLC-based method for identifying ZEN in grains. This selective method could detect fungal-toxin concentrations at the μg/kg level within the limits allowed by European Union and Chilean regulations. Through parameter verification, this method was shown to be suitable for the determination of TCT-B and ZEN contents in grains. By identifying target fungal toxins using HPTLC/MS, the universality and applicability of the method were improved, and the analysis method could serve as a regulatory tool for screening mycotoxins in grains.

### 3.4. Glucocorticoids

Glucocorticoids are a class of endogenous steroid hormones secreted by the adrenal cortex that have anti-inflammatory and anti-allergic pharmacological effects. This type of drug belongs to the S9 category of the WADA’s 2020 prohibition list. Its use in animal husbandry can cause animal protein deposition, which improves the feed-conversion rate and increases economic benefits. Glucocorticoids also play an important role in competition. They promote the synthesis and decomposition of glucose and provide energy to the muscles. However, long-term use of high doses of glucocorticoids may lead to adverse reactions such as immune-system suppression, bone loss, and muscle atrophy.

Detection methods for glucocorticoids in biological samples mainly include the following: TLC, HPLC, GC, GC–MS, LC–MS, and LC–MS/MS. TLC, GC, and HPLC have poor specificity and low sensitivity; thus, their use makes it difficult to meet the requirements for trace veterinary-drug residue analyses (65). GC–MS has a high sensitivity, and many studies have used this method to investigate glucocorticoid residues. Hidalgo et al. (66) detected residual dexamethasone and betamethasone in urine in the negative ion mode in 2003. Compared to GC–MS, LC–MS offers several advantages for the determination of glucocorticoids, including the elimination of the need for derivatization and higher selectivity and sensitivity. Consequently, LC–MS is increasingly replacing GC–MS in this field of analysis. Laboratories in various countries have developed LC–MS and LC–MS/MS techniques to detect glucocorticoids, including ion-trap mass spectrometry, quadrupole mass spectrometry, tandem mass spectrometry, and TOF-MS. Protti et al. (67) introduced a new technique that can detect very low concentrations of glucocorticoids in urine by micro-sampling using dried urine spots (DUS) and volumetric absorption microsampling (VAMS), whereas LC–MS/MS can improve the accuracy and sensitivity of detection. The application of this technology can more effectively detect whether athletes have used prohibited drugs to ensure a fair competitive environment and maintain sportsmanship.

HPLC-Q-TOF-MS has high mass accuracy and resolution and can provide a full-scan analysis of samples with high sensitivity and high mass precision. Liu et al. (58) used HPLC-Q-TOF-MS to analyze and establish a rapid screening method for 15 glucocorticoid residues in pork. HPLC-MS is a commonly used method for trace analysis owing to its high sensitivity and high selectivity. However, the samples must be purified to reduce matrix interference, and traditional SPE pretreatment methods are time-consuming and laborious. The popularity of nanofiber-based solid-phase extraction technology has increased owing to its simplicity, speed, and reduced use of organic solvents (68). A method for detecting 25 types of glucocorticoids, including prednisolone, in milk was established using UPLC-MS/MS, which was employed for testing commercially available milk samples to enrich and purify hormones (69). The analysis of glucocorticoids shows promise using the GC-C-IRMS method. Iannella et al. (70) successfully applied the GC-C-IRMS method to analyze 22 commercially available pharmaceutical preparations containing prednisolone or prednisone, demonstrating its excellent applicability.

## 4. Conclusion

Currently, MS analysis is a widely utilized technique for doping detection due to its exceptional accuracy and sensitivity in detecting minute quantities of stimulants. However, MS-based detection techniques face limitations due to the complexity of the sample pretreatment and separation process, the requirement for a substantial number of samples, the cost of detection equipment, and the time sensitivity of the samples. In this regard, HRMS has effectively addressed these issues and is extensively employed for rapid screening of veterinary-drug residues. At the same time, HRMS has also played a certain driving role in the development of sample pre-treatment technology. The sample processing stage is no longer limited to traditional SPE and liquid–liquid extraction. The pre-treatment process is developing towards simple, fast, and low-cost operations. For example, the pretreatment of GC samples can be cumbersome due to the need for derivatization, while LC–MS is increasingly becoming the preferred method for qualitative and quantitative analysis; the QuEChERS method, as a relatively environment-friendly treatment technology, reduces sample operations, avoids loss of target analytes, improves recovery rates, and has been applied to the extraction of different compounds. Nanofiber-based SPE is another promising technique because of its simple operation, high speed, and low organic-solvent consumption. Currently, urine, whole blood, and serum samples are the primary matrices for detection; however, new supplementary matrices, such as DBS (71), DUS (72), and VAMS (71), have been introduced and verified successfully with improved analyte stability and low cost (73). Synthetic two-dimensional chromatography techniques, such as GC × GC and LC × LC, exhibit enhanced separation capabilities and retrospective analysis potential in the field of anti-doping. These techniques enable the acquisition of comprehensive mass-range datasets and improved detection of analytes in complex samples, making them widely applied in current research.

Detection technology for veterinary-drug residues in animal-derived foods is advancing toward high through put and automation; however, no universal methods for sample pretreatment, chromatographic separation, and MS detection exist. Research has focused on the development of better and faster methods to detect a more comprehensive range of veterinary-drugs in meat products. The establishment of fast, simple, and environment-friendly detection methods will promote the widespread use of detection technology in daily laboratory testing, thereby protecting meat-product safety and human health. Given the similarity between metabolites of certain stimulants and natural metabolites, the selection and optimization of detection methods have become highly challenging. Future research should focus on improving the accuracy and sensitivity of detection methods for animal-derived stimulants, including the development of novel technologies and methodologies. In addition, it is crucial to pay attention to positive drug cases both domestically and internationally, improve the control list for animal-derived stimulants, and promptly adjust the prohibited substances list to prevent the emergence of new stimulants, thereby ensuring food safety standards and the well-being of athletes. Countries should strengthen collaboration and communication to collectively address the challenges faced in stimulant detection.

## Author contributions

QZ and YZ wrote the original draft of the manuscript. QZ and HD conducted the search processes. HD reviewed and edited the manuscript. YZ supervised the manuscript. All authors contributed to the article and approved the submitted version.

## Conflict of interest

The authors declare that the research was conducted without any commercial or financial relationships that could be construed as a potential conflict of interest.

## Publisher’s note

All claims expressed in this article are solely those of the authors and do not necessarily represent those of their affiliated organizations, or those of the publisher, the editors and the reviewers. Any product that may be evaluated in this article, or claim that may be made by its manufacturer, is not guaranteed or endorsed by the publisher.

## Supplementary material

The Supplementary material for this article can be found online at: https://www.frontiersin.org/articles/10.3389/fnut.2023.1226530/full#supplementary-material
