# Supplementary material for: Recent progress on the detection of animal-derived food stimulants using mass spectrometry-based techniques
Source: Front Nutr. 2023 Jul 18;10:1226530. doi: 10.3389/fnut.2023.1226530 (PMC10391635; doi:10.3389/fnut.2023.1226530)
Supplement: Supplementary file 1 [file Data_Sheet_1.docx]

Supplementary Material

Recent Progress on the Detection of Animal-Derived Food Stimulants Using Mass Spectrometry-Based Techniques

Qiang Zhang*, Hongying Du, Yingjian Zhang

*** Correspondence:** Yingjian Zhang: zhangyingjian@lfnu.edu.cn

# Supplementary Tables

**Table S1.** Advantages, disadvantages and applications of common screening methods by chromatography-mass spectrometry

| Resolution | Screening Method | Advantages | Disadvantages | Applications |
| --- | --- | --- | --- | --- |
| Low Resolution | GC-QQQ-MS | Short analysis time, broad analyzable components range, accurate quantification, and high sensitivity for highly polar and volatile compounds analysis. | poor qualitative ability | Food Safety, Environmental Monitoring, Toxicology |
|  | LC-QQQ-MS | Fast analysis, broad analyte coverage, precise quantitation, high-sensitivity detection of complex matrices | poor qualitative ability | Biomedicine, Environmental Monitoring, Food Safety |
|  | LC-Qtrap-MS | Short analysis time, many analyzable components, accurate quantification; double qualitative of unknown compounds, accurate qualitative | High instrument cost, relatively slow analysis speed, and requirement of high-purity samples | Biomedical research, environment, food safety, etc. |
| High resolution | GC-Q-TOF-MS | High resolution, high precision, accurate analysis, non-quantitative and quantitative analysis | Inability to analyze non-volatile and macromolecular compounds; requirement of high-temperature separation. | Environmental pollutants, food additives, etc. |
|  | LC-Q-TOF-MS | High selectivity and sensitivity, ability to analyze macromolecular compounds, non-quantitative and quantitative analysis | Limited ability to analyze macromolecular compounds; requires high separation efficiency and separation time. | Biomacromolecules, Drugs, etc. |
|  | LC-IT-TOF-MS | High sensitivity, strong quantitative analysis ability, outstanding advantages in trace analysis, conducive to structural identification of compounds | Low resolution and precision, slow analysis speed | Macromolecular compounds, such as proteins, peptides, etc. |
|  | LC-LIT/ESI-Orbitrap-HRMS | High sensitivity, strong quantitative analysis ability, outstanding advantages in trace analysis, conducive to structural identification of compounds | Inability to analyze macromolecules; susceptibility of ions to cross-interference. | Metabolomics research, drugs, environment, etc. |
|  | LC-Q/Orbitrap-HRMS | Conducive to the analysis of complex samples, can carry out the analysis of macromolecules and high-precision mass determination | Incapability to conduct MSn analysis; low sensitivity and high cost. | Proteomics research, drug, food, environment, etc. |
|  | Mag-MS | High selectivity and sensitivity; good stability, mature technology, strong quantitative ability | High cost for purchase, operation, and maintenance of instrument, complicated operation, slow analysis speed | Geology, environment, archaeology, semiconductor, food, life science, etc. |
|  | FT-ICR-MS | High selectivity and sensitivity; ultra-high resolution and mass accuracy | Expensive, bulky, and complicated to operate | Biomedicine, environment, food, materials science, etc. |
| Abbreviations: GC-QQQ-MS, Gas Chromatography-Triple Quadrupole Tandem Mass Spectrometry; LC-QQQ-MS, Liquid Chromatography-Triple Quadrupole Tandem Mass Spectrometry; LC-Qtrap-MS, Liquid Chromatography-Quadrupole Hybrid Linear Ion Trap Mass Spectrometry; GC-Q-TOF-MS, Gas Chromatography-Quadrupole-Time-of-Flight Mass Spectrometry; LC-Q-TOF-MS, Liquid Chromatography-Quadrupole-Time-of-Flight Mass Spectrometry; LC-IT-TOF-MS, Liquid Chromatography-Ion Trap-Time-of-Flight Tandem Mass Spectrometry; LC-LIT/ESI-Orbitrap-HRMS, Liquid Chromatography-Linear Ion Trap/Electrostatic Field Orbitrap High Resolution Mass Spectrometry; LC-Q/Orbitrap-HRMS, Liquid Chromatography-Quadrupole/Orbitrap High Resolution Mass Spectrometry; Mag-MS, Magnetic Mass Spectrometry; FT-ICR-MS, Fourier transform ion cyclotron resonance mass spectrometry. | | | | |

**Table S2**: Summary of MS Techniques for the Detection of Animal-Derived Performance-Enhancing Drugs

| Performance-enhancing substance | Compounds | Mass-spectrometric technique | Biological matrix/sample | Sample preparation | CU | Country of origin | Reference |
| --- | --- | --- | --- | --- | --- | --- | --- |
| Endogenous steroids | testosterone | UHPLC-MS/MS | serum | 200 μL Serum sample + 25 μL Ismix + 200 μL H_2_O | c | Italy | (1) |
| AAS | 93 anabolic androgenic steroids | GC-MS/MS | dietary supplements | 10.0 ml liquid sample + 100 μL D3-T (IS, 1.0 ng.μL−1) + 10.0 ml deionized H_2_O + 2.0 ml of alka line buffer solution + 6.0 ml methyl tert butyl ether (TBME) + 6.0 ml n-hexane | c | China | (2) |
| AAS | testosterone | LC-IM-MS | urine | 1 ml urine + 1 ml methanol + 1ml H_2_O + 1 ml methanol | no | USA | (3) |
| AAS | testosterone and nandrolone | GC-MS | urine | 300 pmol ssDNA + 100 μL of selection buffer | c | spain | (4) |
| Pseudo-endogenous steroids | 1-androstene-steroids, 6α-hydroxy-androstenedione, and androstatrienedione | HPLC-IRMS | urine | 10 and 20 ml urine + 2 ml of MeOH + 2 ml H_2_O | no | Germany | (5) |
| AAS | DHCMT | LC-HRMSMS | urine | 250 μl of urine+ 250 μl MQ + 15 μl IS solution | c+f | Austria | (6) |
| AAS | methenolone | HPLC-MS/MS | animal-derived food | 2.0 g of each homogenate+100 μL of d3-methyltestosterone+50 μL of β-glucuronidase/arylsulfatase solution + 5 ml sodium acetate | no | China | (7) |
| Glucocorticoids | 39 glucocorticoids | LC-HRMS | animal-derived food | 2.0 g of each homogenate + 50.0 ml polypropylene centrifuge + 20.0 ml acetonitrile + 5.0 g anhydroussodium sulfate | no | China | (8) |
| AAS | 17 anabolic androgenic steroids | HPLC-MS/MS | dietary supplements | collagen peptide + Musk analgesic aerosol | no | China | (9) |
| AAS | 28 target compounds | GC- MS/MS | dietary supplements | 500 μL of liquid sample + 50 ml polypropylene + 10 ml solvent mixture (MeOH/H_2_O, 50:50, v/v). | no | Italy | (10) |
| AAS | 21 steroids | HRMS | urine | a mixture of MSTFA, NH4I, and 2-mercapto-ethanol +2 μL of each of the 136 available standards solutions of the targetsteroids+50 ml derivatizing agent | no | Italy | (11) |
| AAS Glucocorticoids | 16 glucuronides and 29 sulfate conjugated metabolites | LC-HRMS/(MS) | urine | 2 ml methanol + 2 ml H_2_O + 5ml urine | no | Greece | (12) |
| AAS | methoxyisoflavone and ipriflavone | GC- MS/MS | urine | 2 ml urine + 0.5 ml carbonate/bicarbonate buffer+ 5 ml tert-buthylmethylether | c | Italy | (13) |
| AAS | mesterolone and metenolone | GC-LE-EI-QTOF-MS | urine | 0.5 ml Urine + 25 μL IS-S + 1 ml pH 9.5 buffer 200 mg + NaCl 4 ml EtAc | c | Belgium | (14) |
| AAS | 17 anabolic androgenic steroids | LC-MS/MS | DUS | 30 μL urine sampled (1.03 mg/μL) | c+f | Italy | (15) |
| Glucocorticoids | cortisol, corticosterone, cortisone，dexamethasone, methylprednisolone and fludrocortisone | LC-MS/MS | urine | 100 μl urine + 200 μl sodium acetate buffer + 100 μl H_2_O | no | Italy | (16) |
| AAS | testosterone | IRMS | urine | 2.5 ml urine + 0.5 ml potassium phosphate buffer + 25 μl β-glucuronidase | no | USA | (17) |
| AAS | 28 anabolic androgenic steroids | QuEChERS LC-MS/MS , LC-Q-TOF-MS | animal-derived food | 1 g sample + 0.1% formic acid + 50 ml acetonitrile | c+f | Korea | (18) |
| glucocorticoids | prednisolone and prednisone pharmaceutical formulations | GC-C-IRMS | urine | 10 or 25 ml urine + 1.5 ml phosphate buffer + 100 μL β-glucuronidase | no | Italy | (19) |
| AAS | Stanozolol | HPLC-HRMSMS | urine | 0.5 ml urine + 0.5 ml MQ + 30 μL IS | no | Austria | (20) |
| AAS | Steroid imidazole carbamate derivatives | LC-MS/MS | urine | 2.5 ml urine + 20 μL ISTD + 1ml phosphate buffer | c | Greece | (21) |
| EAAS | androsterone, etiocholanolone, testosterone, epitestosterone, dihydrotestosterone, dehydroepiandrosterone, androstenedione and 17a-hydroxyprogesterone | UHPLC-HRMS | serum | 1 ml Serum sample + 100 μL formic acid | no | Sweden | (22) |
| EAAS | 17 compounds | DS-LC-MS | urine | 300 μl urine | c | Belgium | (23) |
| AAS | nandrolone, boldenone, mesterolone, drostanolone, metenolone, metandienone, oxandrolone, and dehydrochloromethyl T | GC-MS/MS, VAMS | DBS | 20-μL DB + 1 ml H_2_O + 2 ml MTBE | c | United Kingdom | (24) |
| glucocorticoids | prednisone and prednisolone | GC-C-IRMS | urine | 25 or 10 ml urine + 1.5 mlphosphate buffer + 100 μL β-glucuronidase | no | Italy | (25) |
| AAS | metenolone acetate, mesterolone, 17α-methyltestosterone | GC-MS | urine | 2 ml urine + 1.0 ml carbonate buffer + 200 mg NaCl + 4 ml of EtAc | c | Belgium | (26) |
| Abbreviations: AAS, anabolic-androgenic steroids; CU, clean-up; c, centrifugation; f, filtration; DHCMT, dehydrochloromethyltestosterone; DBS, dried blood spot; DS-LC-MS, dilute-and-shoot LC-MS; DUS, dried urine spots; EAAS, endogenous anabolic androgenic steroids; HPLC, high-performance liquid chromatography; HRMS, high-resolution mass spectrometry; HRMSMS, high-resolution tandem mass spectrometry; IRMS, isotope ratio mass spectrometry; GC-LE-EI-QTOF-MS, gas chromatography- low energy electron ionization- quadrupole time-of-flight mass spectrometry; LC-IM-MS, liquid chromatography-ion mobility-mass spectrometry; LC-Q-TOF/MS, liquid chromatography-quadrupole time-of-flight/mass spectrometry; QuEChERS, quick, easy, cheap, effective, rugged, and safe; UHPLC, ultra-high performance liquid chromatography; VAMS; volumetric absorptive microsampling. | | | | | | | |

**Table S3.** Classes of prohibited substances given by the WADA

| Class | Sub-group | Examples |
| --- | --- | --- |
| S0 | Non-approved substances | Any pharmacological substance not addressed by any governmental regulatory health authority |
| S1 | Anabolic androgenic steroids | 1-androstenediol, 1-testosterone, 19-norandrostenediol, bolasterone, nandrolone, stanazolol, methylclostebol |
|  | Other anabolic agents | Clenbuterol, SARMs like tibolone, andraine, zeranol, ligandro |
| S2 | HIF activating agents | Daprodustat, roxadustat, xenon, cobalt, molidustat |
|  | Erythropoiesis stimulants | EPO, darbepoetin |
|  | Chronic gonadotropin and luteinizing hormone | Buserelin, gonadorelin, nafarelin and triptorelin |
|  | Corticotrophins | Corticorelin |
|  | GH, IGF-I, MGF | AOD-9604, hGH 176-191, anamorelin, ipamorelin, GHRPs |
|  | GATA inhibitors | K-11706 |
|  | TGF-β blockers | Luspatercept, sotatercept |
| S3 | β-2 agonists | Fenoterol, formoterol, higenamine, salbutamol, salmeterol, procaterol, vilanterol, arformoterol, levosalbutamol |
| S4 | Aromatase inhibitors | Exemestane, formestane, letrozole, anastrozole |
|  | SERMs | Bazedoxifene, raloxifene, tamoxifen, ospemifene |
|  | Anti-estrogens | Clomifene, cyclofenil, fulvestrant |
|  | Metabolic modulators | AMPK, PPARδ agonist |
| S5 | Diuretics and masking agents | Acetazolamide, amiloride, bumetanide, spironolactone, desmopressin, probenecid |
| S6 | Stimulants | Benfluorex, adrafinil, cocaine, fenfluramine, cathine, ephedrine, octodrine, selegiline |
| S7 | Narcotics | Buprenorphine, methadone, morphine, fentanyl, pentazocine |
| S8 | Cannabinoids | Hashish, marijuana, THCs |
| S9 | Glucocorticoids | Betamethasone, budesonide, hydrocortisone, triamcinolone |
| Abbreviations: AMPK, activators of the AMP-activated protein kinase; EPO, erythropoietin; GH, growth hormone; GHRP, growth hormone releasing peptides;hGH, human growth hormone; HIF, hypoxia-inducible factor; IGF-I, insulin-like growth factor; MGF, mechano growth factors; PPAR, peroxisome proliferator activated receptor δ; SARMs, selective androgen receptor modulators; SERM, selective estrogen receptor modulator; TGF-β, transforming growth factor β; THC,tetrahydrocannabinol. | | |

**References**

1. Ponzetto F, Parasiliti-Caprino M, Gesmundo I, Marinelli L, Nonnato A, Nicoli R, Kuuranne T, Mengozzi G, Ghigo E, Settanni F. Single-run UHPLC-MS/MS method for simultaneous quantification of endogenous steroids and their phase II metabolites in serum for anti-doping purposes. *Talanta* (2023) 255: doi: 10.1016/j.talanta.2022.124218

2. Zhang Y, Wu X, Wang W, Huo J, Luo J, Xu Y, Lu J. Simultaneous detection of 93 anabolic androgenic steroids in dietary supplements using gas chromatography tandem mass spectrometry. *J Pharm Biomed Anal* (2022) 211: doi: 10.1016/j.jpba.2022.114619

3. Velosa DC, Rivera ME, Neal SP, Olsen SSH, Burkus-Matesevac A, Chouinard CD. Toward Routine Analysis of Anabolic Androgenic Steroids in Urine Using Ion Mobility-Mass Spectrometry. *J Am Soc Mass Spectrom* (2022) 33:54–61. doi: 10.1021/jasms.1c00231

4. Shkembi X, Botero ML, Skouridou V, Jauset-Rubio M, Svobodova M, Ballester P, Bashammakh AS, El-Shahawi MS, Alyoubi AO, Sullivan CKO. Novel nandrolone aptamer for rapid colorimetric detection of anabolic steroids. *Anal Biochem* (2022) 658: doi: 10.1016/j.ab.2022.114937

5. Piper T, Thevis M. Addressing recent challenges in isotope ratio mass spectrometry: Development of a method applicable to 1-androstene-steroids, 6 alpha-hydroxy-androstenedione, and androstatrienedione. *Drug Test Anal* (2022) 14:1891–1903. doi: 10.1002/dta.3361

6. Goeschl L, Gmeiner G, Gaertner P, Steinacher M, Forsdahl G. Detection of DHCMT long-term metabolite glucuronides with LC-MSMS as an alternative approach to conventional GC-MSMS analysis. *Steroids* (2022) 180: doi: 10.1016/j.steroids.2022.108979

7. Zheng J, Ye C, Wang P, Liu Y, Yang H, Liu H. Quantitative analysis of total methenolone in animal source food by liquid chromatography-tandem mass spectrometry. *Drug Test Anal* (2021) 13:148–155. doi: 10.1002/dta.2915

8. Yan Y, Ai L, Zhang H, Kang W, Zhang Y, Lian K. Development an automated and high-throughput analytical platform for screening 39 glucocorticoids in animal-derived food for doping control. *Microchem J* (2021) 165: doi: 10.1016/j.microc.2021.106142

9. Wang H, Wang P, Zhao X, Ye C, Zheng X, Cao W. Determination of anabolic androgenic steroids in dietary supplements and external drugs by magnetic solid-phase extraction combined with high-performance liquid chromatography-tandem mass spectrometry. *J Sep Sci* (2021) 44:1950–1960. doi: 10.1002/jssc.202001062

10. Micalizzi G, Huszti K, Palinkas Z, Mandolfino F, Martos E, Dugo P, Mondello L, Utczas M. Reliable identification and quantification of anabolic androgenic steroids in dietary supplements by using gas chromatography coupled to triple quadrupole mass spectrometry. *Drug Test Anal* (2021) 13:128–139. doi: 10.1002/dta.2929

11. Leogrande P, Botre F, de la Torre X, Jardines D, Parr MK, Marini F. Coupling high-resolution mass spectrometry and chemometrics for the structural characterization of anabolic-androgenic steroids and the early detection of unknown designer structures. *Talanta* (2021) 227: doi: 10.1016/j.talanta.2021.122173

12. Kiousi P, Fragkaki A, Kioukia-Fougia N, Angelis Y. Liquid chromatography-mass spectrometry behavior of Girard’s reagent T derivatives of oxosteroid intact phase II metabolites for doping control purposes. *Drug Test Anal* (2021) 13:1822–1834. doi: 10.1002/dta.3056

13. Iannone M, Botre F, Martinez-Brito D, Matteucci R, de la Torre X. Development and application of analytical procedures for the GC-MS/MS analysis of the sulfates metabolites of anabolic androgenic steroids: The pivotal role of chemical hydrolysis. *J Chromatogr B.* (2020) 1155: doi: 10.1016/j.jchromb.2020.122280

14. Albertsdottir AD, Van Gansbeke W, Van Eenoo P, Polet M. Enabling the inclusion of non-hydrolysed sulfated long term anabolic steroid metabolites in a screening for doping substances by means of gas chromatography quadrupole time-of-flight mass spectrometry. *J Chromatogr A* (2021) 1642: doi: 10.1016/j.chroma.2021.462039

15. Protti M, Marasca C, Cirrincione M, Sberna AE, Mandrioli R, Mercolini L. Dried Urine Microsampling Coupled to Liquid Chromatography-Tandem Mass Spectrometry (LC-MS/MS) for the Analysis of Unconjugated Anabolic Androgenic Steroids. *Molecules* (2020) 25: doi: 10.3390/molecules25143210

16. Protti M, Mandrioli R, Mercolini L. Microsampling and LC-MS/MS for antidoping testing of glucocorticoids in urine. *Bioanalysis* (2020) 12:769–782. doi: 10.4155/bio-2020-0044

17. Nair VS, Husk J, Miller GD, van Eenoo P, Crouch A, Eichner D. Evaluation of longitudinal steroid profiling with the ADAMS adaptive model for detection of transdermal, intramuscular, and subcutaneous testosterone administration. *Drug Test Anal* (2020) 12:1419–1431. doi: 10.1002/dta.2885

18. Lee JH, Han JH, Min AY, Kim H, Shin D. Screening for twenty-eight target anabolic-androgenic steroids in protein supplements using QuEChERS extraction followed by liquid chromatography-tandem mass spectrometry. *Food Addit Contam Part A.* (2020) 37:1425–1436. doi: 10.1080/19440049.2020.1773543

19. Iannella L, Botre F, Colamonici C, Curcio D, Ciccarelli C, Mazzarino M, de la Torre X. Carbon isotopic characterization of prednisolone and prednisone pharmaceutical formulations: Implications in antidoping analysis. *Drug Test Anal* (2020) 12:1587–1598. doi: 10.1002/dta.2876

20. Goeschl L, Gmeiner G, Enev V, Kratena N, Gaertner P, Forsdahl G. Development and validation of a simple online-SPE method coupled to high-resolution mass spectrometry for the analysis of stanozolol-N-glucuronides in urine samples. *Drug Test Anal* (2020) 12:1031–1040. doi: 10.1002/dta.2805

21. Fragkaki AG, Petropoulou G, Athanasiadou I, Kiousi P, Kioukia-Fougia N, Archontaki H, Bakeas E, Angelis YS. Determination of anabolic androgenic steroids as imidazole carbamate derivatives in human urine using liquid chromatography-tandem mass spectrometry. *J Sep Sci* (2020) 43:2154–2161. doi: 10.1002/jssc.202000036

22. Elmongy H, Masquelier M, Ericsson M. Development and validation of a UHPLC-HRMS method for the simultaneous determination of the endogenous anabolic androgenic steroids in human serum. *Journal of Chromatography A* (2020) 1613: doi: 10.1016/j.chroma.2019.460686

23. De Wilde L, Roels K, Van Renterghem P, Van Eenoo P, Deventer K. Steroid profiling in urine of intact glucuronidated and sulfated steroids using liquid chromatography-mass spectrometry. *J Chromatogr A* (2020) 1624: doi: 10.1016/j.chroma.2020.461231

24. Chang WC-W, Cowan DA, Walker CJ, Wojek N, Brailsford AD. Determination of anabolic steroids in dried blood using microsampling and gas chromatography-tandem mass spectrometry: Application to a testosterone gel administration study. *J Chromatogr A* (2020) 1628: doi: 10.1016/j.chroma.2020.461445

25. Iannella L, Botre F, Colamonici C, Curcio D, de la Torre X. Development and validation of a method to confirm the exogenous origin of prednisone and prednisolone by GC-C-IRMS. *Drug Test Anal.* (2019) 11:1615–1628. doi: 10.1002/dta.2715

26. Albertsdottir AD, Van Gansbeke W, Van Eenoo P, Polet M. Evaluation of alternative gas chromatographic and mass spectrometric behaviour of trimethylsilyl-derivatives of non-hydrolysed sulfated anabolic steroids. *Drug Test Anal.* doi: 10.1002/dta.346
